# Community-based interventions for mitigating cognitive-linguistic decline in older adults

**DOI:** 10.3389/fpsyt.2024.1422037

**Published:** 2024-10-03

**Authors:** Xiaoming Tian, Wenshu Ding

**Affiliations:** School of Humanities and Foreign Languages, Xi’an University of Posts and Telecommunications, Xi’an, China

**Keywords:** older adults, cognitive-linguistic health, rehabilitation, rural areas, interventions

## Abstract

**Introduction:**

This study focuses on exploring the effectiveness of interventions in two community-based language rehabilitation care centers located in an economically underdeveloped region of northwest China.

**Method:**

The research focuses on 35 older adults with cognitive-linguistic decline enrolled in both centers. The interventions provided to the participants include auditory training, sign language use enhancement, second language interest classes, and the promotion of elderly-mode applications on smartphones.

**Results:**

The interventions were particularly effective in enhancing auditory processing and language comprehension skills. Additionally, the promotion of elderly-mode applications on smartphones proved to be a valuable tool in improving participants’ language cognition.

**Conclusion:**

The study underscores the importance of tailored community-based interventions in improving language skills and cognition and provides a basis for further research in this area.

## 1 Introduction

China is currently facing the dual challenges of a notable deceleration in population growth and the predicament of an aging population (1). The seventh Chinese population census in 2020 revealed that 18.70% of China’s total population was aged 60 years and older, with 13.50% aged 65 years and older (3). Projections suggest that by 2025, the population of individuals aged 60 and above in China will reach 300 million, categorizing it as a super-aging nation (2). Consequently, research about issues affecting the elderly has become increasingly urgent. Among these concerns, the study of language aging and its underlying mechanisms has garnered significant attention from the scholarly community (3). Such research is integral to comprehending aspects like human language development and cognitive changes in the brain (3).

The term *gerolinguistics*, first introduced by Cohen in 1979, has since spurred numerous studies on the verbal communication of older adults, language impairment, and cognitive aging, as well as the sign language use of older adults (4). Cognition often refers to the process by which the human brain assimilates information from the external environment and acquires knowledge through mental activities such as conceptualization, perception, memory, judgment, and imagination (5). As individuals age, there are changes in the neural substrates that may lead to a decline in cognitive resources such as processing speed, working memory, breadth of attention, and rehabilitation (5). Alterations in cognitive function are indicative of physiological aging and are closely linked to changes in the neural structure and function of the brain (6). Conditions such as aphasia, Alzheimer’s disease, and other types of cognitive disorders can adversely impact the physical and mental health of the elderly, causing distress to their families (6). Thus, there is a pressing need to prevent and ameliorate the cognitive-linguistic decline in the elderly.

Nevertheless, the development of rehabilitation for speech and language cognitive disorders among older adults remains limited in China (7). Institutions providing speech and language rehabilitation services are predominantly general or rehabilitation hospitals (7). While hospitals offer advantages such as rapid bed turnover and short treatment cycles, they are ill-equipped to sustain the effectiveness of cognitive-linguistic disorders requiring long-term interventions (7). Furthermore, with the advancement of China’s socialist modernization, industrialization and urbanization are accelerating, leading to a significant migration of young people from rural areas and small towns to urban centers. This demographic shift has resulted in a growing number of ‘empty nesters’ in rural areas and small towns (8). The long-term absence of companionship from children and grandchildren, coupled with dispersed living arrangements and inadequate social organization, often leaves the elderly feeling isolated, diminishing their opportunities for language communication, and resulting in speech impairments such as reticence, slurred speech, and stuttering. These issues are indicative of the decline in the language ability of older adults, significantly impacting their standard of living and quality of life (8).

Presently, community-based elderly care centers in China, particularly those situated in remote or economically underdeveloped areas, encounter challenges in providing language rehabilitation services to the elderly due to a lack of personnel with relevant professional backgrounds, therapeutic equipment, and suitable consultation rooms, despite the evident demand for such services (9). Notably, the small county where we conducted part-time work hosts two community-based language rehabilitation care centers, despite being in an economically underdeveloped region. The two centers provided us with an opportunity to engage in cognitive-linguistic interventions.

Our involvement as part-time staff at both centers – one of us is a researcher specializing in cognitive linguistics for older adults, and the other a volunteer working in the field of older people’s cognitive-linguistic health – involved providing language rehabilitation service to the 35 older adults from the two centers. Our efforts focused on studying the deterioration of language cognition in the older adults and endeavoring to enhance their quality of life through appropriate interventions and services. Consequently, this paper integrates theories of cognitive-linguistic decline in older adults with practical interventional experiences in language rehabilitation service delivery to delineate effective intervention practices for cognitive-linguistic decline. Additionally, we reflect on shortcomings and offer recommendations, considering intervention practices and feedback from field trials.

## 2 Context

This study focuses on a small, relatively remote county in northwest China, where the two community-based language rehabilitation care centers are located. The county, nestled amidst mountainous terrain and a vast Gobi Desert, experiences a dry climate, covers a vast area, and has a low population density. In comparison to the more developed eastern seaboard, it lags in terms of economic development and lacks adequate medical resources. Approximately 30 years ago, the county witnessed economic prosperity due to its abundant natural resources, particularly coal mines. However, with the depletion of these resources, economic growth has stagnated, leading to a significant exodus of young people seeking work opportunities in more economically developed regions of China. Presently, the county’s GDP ranks among the lowest within its province, and the lingering impact of this economic decline is still palpable among the local population, despite the economic boom being a distant memory.

The field survey conducted for this study revealed that older adults in the region often fondly reminisce about their youth. These elderly individuals, who were once considered pioneers of fashion in China, enjoyed the benefits of the well-developed local economy. They were among the first in the country to own televisions and sewing machines, long before such household appliances became commonplace nationwide. Moreover, while much of the country was still grappling with economic hardships, the residents of this county were already focusing on spiritual and cultural development. In the 1990s, a time when disco music was just beginning to gain popularity in major cities across China, several disco venues were established in this small county. Today, remnants of this era, such as old concert halls and billboards with the word ‘disco’ in English, serve as reminders of the region’s past prosperity, despite many of these establishments now being defunct. The target population of this study comprises older adults in the county with language cognitive impairment or decline.

## 3 Methods

At the time of the research, the two community-based centers participating in this study were providing language rehabilitation services to 35 older adults with cognitive-linguistic impairment or decline. Through professional interventions, alongside feedback from field trials, the study implemented a series of rehabilitation and intervention activities targeting the impairment and decline in language function among these older adults. These activities included optimizing auditory training through enhancing sign language use, offering second language interest classes, and promoting the use of elderly-mode applications on smartphones.

The primary participants of the study, comprising 35 older adults residing in the two centers, are all aged 60 years or older. This specific group typically exhibits the following characteristics: they are often ‘empty nesters,’ with their children having moved away for work; they have received a relatively comprehensive secondary education, which is uncommon among the elderly in China; they use Standard Mandarin (Putonghua) in their daily lives; they receive a stable pension income; they are open to embracing new ideas, technologies, languages, and cultures; they have normal intellectual development, although some may have a history of chronic diseases; they are physically capable of self-care, though they exhibit varying degrees of cognitive-linguistic decline.

The interventions lasted for eight weeks and were conducted through three major activities. The first activity focused on auditory training and the use of sign language. This activity was conducted eight times, with one session per week, each lasting one hour. During the first half-hour of each session, the older adults were trained in auditory skills and the use of sign language. The second half-hour involved pair or group conversations to practice these skills, allowing both researchers to observe their sign language use and record significant changes in their audio-visual abilities. The second activity involved teaching basic English words to develop an interest in the English language. This activity was also conducted eight times, with one session per week, each lasting one hour. The first half-hour of each session was dedicated to teaching basic English words commonly encountered in daily life, such as ‘WiFi,’ ‘App,’ and ‘KFC.’ In the second half-hour, pictures or demonstrations featuring both Chinese and English were used to assess the extent to which the participants could understand some English words in a bilingual context. The third activity focused on promoting the use of elderly-mode applications on smartphones. This activity, like the others, was conducted eight times, with one session per week, each lasting one hour. The first half-hour involved instruction on using elderly-mode applications, with demonstrations and hands-on practices. In the second half-hour, life scenario-based tasks, such as calling for first aid, were set up to test the participants’ ability to independently use the applications on smartphones.

The entire intervention process was carried out through a division of labor and cooperation between both researchers. Specifically, one researcher was responsible for organizing the intervention and rehabilitation activities for the 35 older adults at the two centers, while the other researcher was responsible for recording the participants’ performance and feedback through note-taking and diary-keeping. After each intervention activity, the two researchers organized, discussed, and reviewed the recorded data, focusing on the cognitive-linguistic progress of each participant, the specific actions or practices that contributed to this progress, and the barriers to cognitive-linguistic growth encountered during the process. In addition to analyzing the intervention data, the researchers also communicated with other staff members at the two centers to collect additional related data on the participants’ language performance following each intervention. The findings from these activities are reported in the following section.

## 4 Interventions: excellent practices

### 4.1 Enhancing auditory training through sign language use

With advancing age, cognitive functions decline, leading to a gradual decrease in working memory and depletion of cognitive resources, particularly evident in the decline of language competence (10). The interruption phenomenon related to language understanding in older adults may be linked to age-related hearing loss (11). Despite these cognitive changes affecting information reception, older adults demonstrate a comparable ability to understand and make inferences in context compared to younger adults. White and Abrams found no significant difference between older and younger adults in the amount of semantic priming effect, indicating that older adults maintain their ability to understand contextual information and make semantic associations (12).

Semantic priming is an implicit memory process that occurs without the need for subjective attention or conscious involvement (13). Based on this understanding, we implemented a strategy to enhance semantic understanding and inference for the 35 older adults by providing contextual cues for words that may not be heard clearly. For instance, in situations relevant to the older adults, we presented a word with a superordinate meaning first, followed by two words – one associated and one unrelated – to stimulate their implicit memory’s selective capacity. This approach enhances accurate inference of words that were unclear in the context (infra-segmental meaning). For example, in our interventions, when the older adults heard the word ‘family’ first, they recognized the word ‘children’ significantly faster than ‘television,’ indicating a contextual association between ‘family’ and ‘children’.

In practice, we observed that raising the volume or slowing down speech, known as elder-directed speech (elderspeak), is commonly used by language barrier interventionists to facilitate communication with older adults (14). Elderspeak, like child-directed speech, is tailored for a specific age group (14). However, the interventions revealed that while elderspeak may aid in semantic understanding to some extent, some of the older adults in the study viewed it as discriminatory and responded by pretending to be unresponsive. Hence, relying solely on elderspeak is not the most effective strategy for addressing the language recognition issue in the older adults.

In a visual context, the older adults tended to pay more attention to words during reading, with multiple repetitions of gaze points. They relied heavily on semantics and possessed a superior ability to predict vocabulary compared to younger individuals. Our interventions, therefore, focused on enhancing body language, emphasizing emotional changes in tone, and using visual aids like pictures to facilitate faster semantic understanding – proving to be an effective communication strategy for the older adults in this study. For instance, in scenarios discussing a specific body part that was causing discomfort, we deliberately used intonation cues such as ‘right’, ‘this’, and ‘here’ to build context in questioning, or pointed to the chest while saying the word ‘heart’ to ensure quick comprehension.

Retrieval deficit is a key factor affecting reading inference processing in older adults. McGinnis and Zelinski conducted a study comparing inference processing differences between young adults and two cohorts of older adults (15). The study highlighted older adults’ reasoning processing and extraction impairment across age groups (15). These findings support the Abstraction Deficit Hypothesis, suggesting that older adults focus more on the overall viewpoint of a text and overlook conceptual levels of meaning that contribute to specific semantic content comprehension (16).

In addressing extraction disorder characteristics, a strategy of elder-directed speech can be effective for older adults who do not reject speech volume adjustment or rate reduction. As information processing speed decreases with age, older adults with cognitive impairment or decline may experience language production deficits, such as speechlessness or tongue-tiredness, affecting normal communication and potentially endangering their lives and health in emergencies. Older adults’ communication difficulties are not due to a lack of knowledge but rather to an inability to map their thoughts to the corresponding phonological form and retrieve the phonemic representation of words (17). Words susceptible to the tip-of-the-tongue phenomenon are often proper nouns, with low-frequency words more likely to cause this phenomenon (18, 19). To address this, we incorporated synonym or near-synonym substitution in the interventions. For example, when the term ‘quick-acting heart pills’ (in Chinese, suxiao jiuxinwan) seemed unfamiliar to the older adults, we introduced the related term ‘heart disease’ to enhance their understanding and verbal response.

The positive outcomes of these interventions were evident in our field trials. The older adults generally showed improved comprehension and expression of language, as well as increased engagement in communication activities. For example, during our interventions, we observed that some of the older adults were more willing to participate in discussions and activities when provided with contextual cues and visual aids. Additionally, their ability to recall and use new vocabulary improved, indicating a positive impact on their language cognition.

Furthermore, these interventions not only improved language cognitive abilities but also enhanced the overall quality of life for the older adults. Improved communication skills led to better social interactions and reduced feelings of isolation and loneliness. Some of the older adults reported feeling more confident in their ability to communicate and engage with people around them, leading to increased participation in social activities and a greater sense of well-being.

### 4.2 Providing interest-based second language classes

Existing studies have highlighted the unique cognitive characteristics exhibited by bilingual or multilingual learners during the aging process compared to monolinguals (20). Rossi and Diaz provided insights into the effects of aging and bilingual experience on language processing, suggesting that bilingual children outperform monolingual ones in both language output and comprehension (21). This superiority is attributed to the continuous activation and inhibition of both languages, leading to the development of superior inhibitory abilities (21). Inhibition refers to the conscious control of irrelevant information while performing a task (22). During language processing, bilinguals are more likely to activate brain regions such as the dorsolateral prefrontal cortex, anterior cingulate gyrus, and basal ganglia (16). Long-term bilingual use and monitoring result in functional and structural changes in the brains of bilinguals, further enhancing their executive control (23). Another study supported this idea, suggesting that bilinguals delay the onset of Alzheimer’s disease by enhancing executive control in the frontal striatum and frontal-parietal brain regions (24). Experimental studies have also shown that bilingual training for both young and older individuals can improve cognitive flexibility and enhance pre-control before language processing (25).

Given the increasing prevalence of foreign language use in everyday life due to globalization, second language learning for older adults is particularly important. It not only improves their language processing inhibition but also fills in any ‘knowledge blind spots’ that may exist. A two-month second language teaching program was conducted in the two centers, focusing on teaching vocabulary commonly used by the elderly in their daily lives, including pronunciation, morphology, and specific meanings. For example, understanding terms such as ‘WiFi’ and ‘WLAN’ became crucial as older adults may need to connect to the internet or switch channels when using television. In the second language interest classes offered as part of the interventions, we focused on teaching English words related to television and mobile phone use. We conducted listening and reading exercises for the word ‘APP,’ explained its Chinese meaning, and placed it in common contexts, such as ‘download an APP’ or ‘update an APP.’ Similarly, words like ‘WLAN’ and ‘WiFi’ were included in the teaching process. However, due to the abstract nature of these terms and the potential lack of background knowledge among the older adults, there were challenges in the teaching process, particularly in choosing interpretations.

In contrast, our interventions found that food-related English words were easier for the older adults to grasp. For example, when learning the word ‘coffee’, tasting it first helps them understand and remember it, and the phonetic translation of ‘coffee’ makes it easier for them to understand and pronounce. At the same time, we introduced coffee brands such as ‘Starbucks’ and ‘Costa’ to them, but given the influence of culture and values, most of them thought that these brands of coffee were too expensive and preferred to drink tea. This suggests that when introducing something new, the cultural background and values of the elderly should be fully considered to minimize possible misunderstanding and confusion.

These language learning interventions had several positive outcomes. Firstly, the older adults showed improved cognitive abilities, particularly in terms of language processing inhibition and executive control. They were able to comprehend and use new vocabulary more effectively, enhancing their communication skills. Secondly, the interventions increased social engagement between the older adults and the young people around them, as they gained confidence in understanding and using some English words familiar to the younger generation. This, to some extent, improved their overall quality of life and helped reduce feelings of isolation and loneliness. Lastly, the interventions helped older adults adapt to the changing linguistic landscape, particularly in a globalized world where foreign language use is becoming more prevalent.

### 4.3 Encouraging the adoption of elderly-mode applications on smartphones

As individuals age, there is a natural decline in cognitive functions, including a gradual decrease in working memory and cognitive resources, particularly in verbal cognition, which exhibits a significant downward trend (10). This decline often leads to older adults becoming more susceptible to interference from irrelevant salient information, resulting in a weakening of their executive control ability. Executive control encompasses coordinating and controlling cognitive operations, such as inhibitory control (selecting relevant information for processing and inhibiting interference from irrelevant information), attention conversation, and refreshing working memory (26).

To address the challenges faced by some of the older adults in the study, we implemented a comprehensive program to promote the use of elderly-mode applications on smartphones in the two centers, aiming to enhance their overall user experience and facilitate smart aging. The elderly mode was meticulously designed to incorporate features such as short, gentle, and soothing alert tones, along with visual aids such as bold Chinese characters for easier readability. This design choice aimed to maintain a clear and concise interface, thereby avoiding information overload, and improving the effectiveness of information perception among older users (15).

As part of the interventions, our efforts were directed toward familiarizing the older adults with various elderly-mode applications on smartphones that they use in their daily lives. This included guiding them on making phone calls, checking weather forecasts, and using mobile payment services. Furthermore, we placed a strong emphasis on enhancing their phone-dialing skills and understanding their critical role in emergencies. Additionally, we assisted each of the 35 older adults in setting up an emergency contact, prioritizing their health and safety. These efforts were crucial in empowering the older adults to navigate the digital world with confidence and independence.

The positive outcomes of the interventions became increasingly evident as the older adults began to use some of the elderly-mode applications on their smartphones. One notable example was the significant improvement in their ability to utilize the emergency contact function. This was demonstrated when one of the older adults, who had previously struggled with this feature, successfully contacted emergency services during a heart attack. The timely arrival of medical assistance underscored the practical benefits of the interventions and highlighted its potential to enhance the safety and well-being of older adults.

However, we also faced some challenges. One notable issue was the resistance from some of the 35 older adults toward the term ‘elderly mode,’ which they perceived as age-discriminatory. In response, we recommend that software designers adopt a more inclusive and welcoming term, such as ‘caring mode’ or ‘age-friendly mode,’ to better reflect the compassionate intent and positive impact of the mode.

## 5 Discussion

### 5.1 Highlighting self-esteem in language rehabilitation services

When guiding the older adults to use sign language, we encountered various challenges related to their self-esteem. Some of them exhibited aversion, perceiving it as a negative reflection on their intelligence or cognitive abilities (27). This reaction sometimes led them to pretend not to understand, which posed a significant challenge to the intervention efforts. A similar phenomenon was observed in the promotion of elderly-mode applications on smartphones. Some participants exhibited a naturally negative attitude toward age-related terms such as ‘elderly phone’ or ‘elderly mode,’ often hesitating to discuss or use them voluntarily. Some even refused to learn or adopt them, citing concerns about the stigma associated with aging.

To solve the problem and enhance the interventions, we implemented several strategies. First, following previous research (28), we revised our language to be more respectful and empowering, replacing terms like ‘elderly mode’ with ‘accessibility mode,’ which was more acceptable to the older adults. Additionally, building on the literature (29), rather than highlighting how the elderly-mode applications differ from standard ones, we focused on the practical benefits such as enhancing independence, connectivity, and convenience. By presenting these applications as tools for empowerment, we reduced resistance among the older adults. Furthermore, we encouraged peer support and assistance during the learning process, creating a supportive environment where the participants could share experiences, and fostering a sense of community. This approach boosted self-esteem and confidence, motivating them to engage with new technologies more enthusiastically.

### 5.2 Addressing educational variance and misconception

Researchers proposed a structural model of cognitive aging highlighting that cognitive processing speed is influenced by both age and level of education (30). In the interventions, most of the older adults had a secondary education, while few had a higher education background. This educational variance likely contributed to difficulties in comprehending abstract concepts. For example, when assessing the participants’ understanding of Internet TV, terms like ‘WiFi’, ‘network’, ‘fiber optic’, and ‘router’ proved especially challenging. Many participants had difficulty comprehending the technical aspects of these terms, leading to unclear or delayed responses, a pattern also observed in other research (31). To address this challenge, we explored strategies to present abstract concepts more effectively. One approach we were considering was the use of analogies or real-life examples to illustrate complex ideas. For instance, when explaining the concept of ‘WiFi’, we compared it to a wireless phone connection, emphasizing the absence of physical cables. Similarly, we described a ‘network’ as being like a spiderweb, highlighting the idea of interconnectedness. Additionally, we also used more visual aids, such as diagrams and illustrations, to reinforce the verbal explanations.

Research suggests that education primarily preserves existing cognitive functions rather than directly impacting cognitive aging (32). However, our interventions revealed that many participants, particularly those with higher education, hold significant biases or misconceptions about cancer, often leading to exaggerated emotional reactions upon diagnosis. The prevailing belief among the participants that cancer is incurable contributes to their low acceptance of traditional treatments like radiotherapy and chemotherapy, with some refusing or avoiding medical treatment (33). Since cancer is a leading cause of death among older adults, it is crucial to deepen our understanding of cognitive functioning in this population and implement interventions that promote a more objective understanding of cancer-related information (34). Future research efforts should focus on developing targeted strategies to enhance older adults’ comprehension of health information, especially regarding cancer.

### 5.3 Cultivating acceptance of new ideas and products

Similar to findings in other research (34), our interventions suggested that many of the participants, like the majority of older adults in China, tended to have a frugal mindset, making them reluctant to adopt new or expensive products. For instance, when introduced to higher-priced imported goods like ‘Costa’ and ‘Starbucks’, some participants struggled to understand their value, leading to skepticism and hesitation in trying these products. This mindset not only impeded their acceptance of new items but also widened the generation gap, as younger generations in China are more receptive to global consumer trends.

To address these challenges, we incorporated the cultural backgrounds of different countries and nationalities into the interventions. Research showed that engaging in cross-cultural and cross-linguistic learning can help broaden the horizons of older adults and increase their openness to new ideas and products (21). For instance, we found that explaining the cultural significance of foods or products from other countries to some extent helped some participants appreciate their value beyond the price. Consistent with the literature (35), we also found that including cultural exchange in interventions can help bridge generational gaps, promoting mutual understanding. Additionally, organizing events where the participants could sample new foods and learn about their cultural significance made unfamiliar items more approachable. Along with other research (36), we found that presenting information in a clear, accessible manner – using simple language and visual aids – enhanced some participants’ understanding of new products, increasing their willingness to adopt them.

## 6 Conclusion

Building on related studies (37), this research proposed a comprehensive set of cognitive-linguistic rehabilitation measures designed to address the physiological and functional characteristics of cognitive impairment and decline in older adults. First, sign language was used to help the older adults better grasp and understand semantic content. Second, synonym and near-synonym substitution techniques were introduced to address the ‘tip-of-the-tongue phenomenon’, which is common in older adults’ language production. Additionally, bilingualism was used as a means to enhance old adults’ inhibitory control during language processing. Finally, to mitigate the decline in executive function with age, the use of elderly-mode applications on mobile phones was promoted, which to a great extent reduced distractions from irrelevant information, optimizing the efficiency of information processing.

However, during the actual inventions, we identified limitations and challenges that need further refinement. These included various factors such as education level, family environment, upbringing, and personal traits. To make cognitive-linguistic rehabilitation more effective, it is essential to take into account the comprehensive physical and psychological characteristics of older adults (38). Only by thoroughly understanding and carefully considering these factors can more substantial and long-lasting rehabilitation outcomes be achieved.

This study not only explored the current state of cognitive-linguistic decline among the older adults in the two research participatory centers but also proposed a series of targeted rehabilitation strategies tailored to their cognitive-linguistic and functional needs. These strategies aimed to help them overcome cognitive decline and improve their language skills and executive control but also targeted to provide them with a more humane and effective rehabilitation experience. Given that language and cognitive aging is a complex process affected by multiple factors, future research should further investigate and validate various intervention approaches and their effectiveness, providing a stronger empirical basis for slowing down cognitive-linguistic impartment and decline in older adults.

## Data Availability

The original contributions presented in the study are included in the article/supplementary material. Further inquiries can be directed to the corresponding author.
